# Mast Cells in the Mammalian Testis and Epididymis—Animal Models and Detection Methods

**DOI:** 10.3390/ijms23052547

**Published:** 2022-02-25

**Authors:** Marta Himelreich-Perić, Ana Katušić-Bojanac, Marko Hohšteter, Nino Sinčić, Vedrana Mužić-Radović, Davor Ježek

**Affiliations:** 1 Scientific Centre of Excellence for Reproductive and Regenerative Medicine, School of Medicine, University of Zagreb, 10000 Zagreb, Croatia; ana.katusic@mef.hr (A.K.-B.); nino.sincic@mef.hr (N.S.); davor.jezek@mef.hr (D.J.); 2 Department of Biology, School of Medicine, University of Zagreb, 10000 Zagreb, Croatia; 3 Department of Veterinary Pathology, Faculty of Veterinary Medicine, University of Zagreb, 10000 Zagreb, Croatia; marko.hohsteter@vef.hr; 4 Hospital for Medical Rehabilitation of the Health and Lung Diseases and Rheumatism “Thalassotherapia-Opatija”, 51410 Opatija, Croatia; vedmuzic@yahoo.com; 5 Department of Histology and Embryology, School of Medicine, University of Zagreb, 10000 Zagreb, Croatia

**Keywords:** mast cells, model, mammal, testis, epididymis

## Abstract

Mast cells (MCs) are an evolutionary well-conserved type of cells, mediating and modulating allergic responses in innate immunity and tissue remodeling after chronic inflammation. Among other tissues, they inhabit both the testis and epididymis. In the testis, MCs usually appear in the interstitial compartment in humans, but not in other standard experimental models, like rats and mice. MCs seem to be responsible for testicular tissue fibrosis in different causes of infertility. Although experimental animal models follow the effect on MC activation or penetration to the interstitial tissue like in humans to some extent, there is an inconsistency in the available literature regarding experimental design, animal strain, and detection methods used. This comprehensive review offers an insight into the literature on MCs in mammalian testes and epididymides. We aimed to find the most suitable model for research on MC and offer recommendations for future experimental designs. When using in vivo animal models, tunica albuginea incorporation and standard histological assessment need to be included. Domesticated boar strains kept in modified controlled conditions exhibit the highest similarity to the MC distribution in the human testis. 3D testicular models are promising but need further fine-tuning to become a valid model for MC investigation.

## 1. Introduction

Mast cells (MCs) have a crucial role in promoting hypersensitivity reactions and reactions to parasitic diseases. They are essential in developing autoimmune diseases, promoting acute and chronic inflammatory responses [1,2], and recognized as critical regulators of immune modulation, capable of suppressing allergic reactions and chronic inflammation [3]. Mast cell precursor population originates at the yolk sac [4], while in adult tissues, MC precursors reside in the bone marrow and migrate to tissues where they further differentiate and serve as sentinel cells under the influence of intrinsic and external stimuli [5]. Mast cell hematopoietic progenitors express CD34+ on their surface, and both KIT (type III receptor tyrosine kinase, CD117) and interleukin (IL) 3 initiate their differentiation in the bone marrow [6,7]. *c-kit*, which encodes for KIT (CD117), is essential in regulating all aspects of MC biology besides differentiation: survival, proliferation, secretory functions, and migration. Unlike MCs, most hematopoietic cells lose their KIT expression in the process of differentiation. The stem cell factor (SCF) functions as its specific ligand and has several other names—steel factor, MC growth factor, or, most commonly, KIT ligand [8]. Its gradient can serve as a MC chemoattractant, thus regulating almost all aspects of MC functions [9].

**Figure 1:**
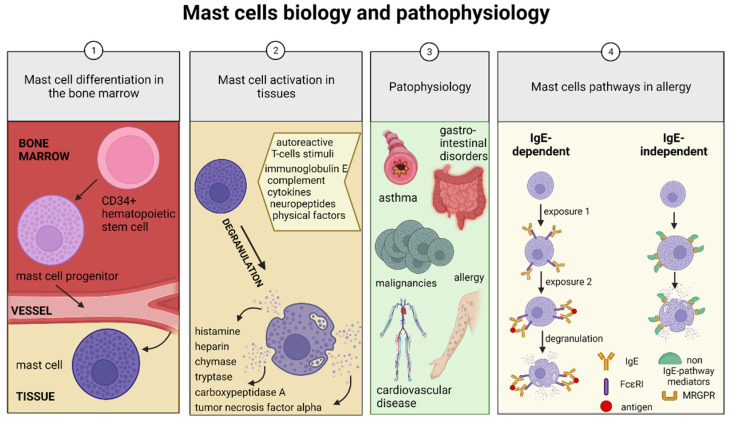
Mast cell (MC) biology and pathophysiology. (**1**) The CD34+ hematopoietic stem cell is the MC precursor differentiating into MC progenitors in the bone marrow. They reach the tissues MCs reside in and differentiate locally. (**2**) Under various stimuli, MCs degranulate, and the secreted mediators affect surrounding cells. (**3**) MCs are crucial in the pathophysiology of asthma, gastrointestinal disorders, allergy, cardiovascular disease, vasodilatation, hematostasis, and cancer. (**4**) MCs can be activated by immunoglobulin (Ig)E-dependent and IgE-independent pathways. IgE-dependent stimulation starts with pre-exposure to an antigen, which sensitizes the MC. The second exposure links the IgE and high-affinity IgE receptor (FcεR1) with the antigen and causes degranulation. The IgE-independent pathway does not require sensitization. Various mediators (neuropeptide Y, substance P, complement fragments polypeptides, cytokines, toxins) can directly activate or degranulate MCs.

After commitment, MCs leave the bone marrow through blood vessels and migrate to various organs with the help of lymphocyte peyer patch adhesion molecule (α4β7 integrin), mucosal addressin cell adhesion molecule-1 (MAdCAM-1), and vascular cell adhesion molecule-1 (VCAM1) (surface molecules) and other molecules that aid their homing in tissues (Figure 1) [10]. MCs mature and differentiate to their final form locally, where they reside scattered around blood vessels and nerve fibers in the mucosa and connective tissue. They inhabit the connective tissue of the skin and of the respiratory and gastrointestinal tracts [11].

MCs are mononuclear, granulated cells of the immune system that have an oval or irregular shape. Due to the presence of acidic histamine, the abundance of granules that overlay the centrally positioned nucleus stain metachromatically with alkaline dyes [12]. Intact mast cells have tightly packed granules; they are spindle-shaped, unlike spreading MCs, which have fewer granules, but both stain purple red with toluidine blue. On the other hand, degranulated cells are pale pink with a prominent nucleus and no longer stain metachromatically [13].

MC activation occurs as a response to autoreactive T-cells stimuli, immunoglobulin E, complement, cytokines, neuropeptides, physical trauma, or sunlight [2]. In the granules of MCs, histamine, heparin, chymase, tryptase, cathepsin G, carboxypeptidase A, and tumor necrosis factor-alpha (TNFα) can be found pre-synthesized and may be released in the surrounding tissue right after MC activation [14]. Consequently, degranulation of existing MC granules and de novo cytokine (TNFα, IL-6, and IL-1β), prostaglandins, and leukotriene production (Figure 1) [15,16] may take place by linking the activating compounds to the receptors in the MC plasma membrane, subsequently affecting vascular permeabilization (short term effect), angiogenesis, and tissue repair (long term effect) [2]. An increase in gene transcription may add to the prolonged MC activation effect [13]. As a result, MCs may influence metabolism, tissue remodeling, inflammation, metaplastic development, reproductive functions, blood clotting, and sleep [17].

The secreted protease content is the essential feature differentiating human populations of MCs: MCTs, which secrete only tryptase, MCCs secrete only chymase, while MCTCs contain tryptase, chymase, carboxypeptidase, and cathepsin [2,18]. In rodents, two types of MCs exist, differing in their location, staining, and protease content: mucosal-type MCs, similar to human MCT, and connective tissue-type MCs, similar to MCTCs [19].

## 2. Mast Cell Disorders

Primary MC disorders include mastocytosis and primary MC activation syndrome (MCAS). The symptoms include flushing, angioedema, diarrhea, abdominal pain, joint hypermobility, hypotensive episodes, and autonomic dysfunction. Mastocytosis is the clonal MC proliferation due to *c-kit* mutation, making MCs prone to proliferation and sensitive to degranulation. MC leukemias and sarcomas are rare forms of systemic mastocytosis [20]. MCs influence inflammation, fibrosis, the gastrointestinal and reproductive tracts, hemostasis, the and cardiovascular system [17], and their disturbances are classified as secondary MC disorders, often influenced by interleukin (IL)-3, IL-4, IL-9, IL-10, IL-33, TGF-β, SCF and many other agents (Figure 1) [21]. In recent years, significantly more is known about their regulatory, physiological function, such as wound healing, immune tolerance, and suppression of allergies, and not just their pathological involvement [3].

### 2.1. Allergies

The functional characterization of MCs is complex due to their distribution, but also to their dual behavior in the organism since they can act simultaneously as “sensors” [22] and effective “warriors”.

Two major routes of MC activation are known—immunoglobulin E (IgE)-dependent and IgE-independent pathways (Figure 1). The IgE-dependent pathway is considered as the main one for MC physiological activation in host defense against parasitic infections and the initiation of type I allergic reactions [23] and requires sensitization to an allergen. On the other hand, IgE-independent pathways have also been proven to serve pivotal roles in the pathophysiology of allergic and pseudoallergic responses but include MC activation by inflammatory mediators, complement fragments, cytokines, and neuropeptide substance P through specific G-protein-coupled receptors (GPCR) [3].

When activated by IgE-induced signaling through the canonical high-affinity IgE receptor (FcεRI) [23], MCs respond through an active degranulation process, characterized by a fast release of various intracellularly-stored mediators. The inducers and tissue-specific supporters of MC active response remain incompletely characterized, despite recently proposed candidates. One of them is interleukin IL-33, a constitutively expressed IL-1 family member, having above mentioned the dual role of activation and support of MCs with a significant emphasis on their inflammatory response [22].

IL-10 too has a dual role that may contribute to a negative feedback regulation in the context of inflammation-related pathologies, in which IL-10 promotes the transient expansion of MCs but then terminates the inflammatory milieu by the induction of MC apoptosis [24].

IgE-independent signaling pathways have been related to MC activity in immediate hypersensitivity reactions after the discovery that a diverse range of peptides such as neuropeptide Y or substance P [25], nerve growth factor (NGF), calcitonin gene-related peptide (CGRP), and pituitary adenylate cyclase-activating peptide and platelet-activating factor (PAF)-4 [26] can activate human MC through the members of the G protein-coupled receptor family (GPCR), called Mas-related G protein-coupled subfamily of receptors (MRGPRs). Notably, MC membrane receptor MRGPRX2 has been identified as a cause of pseudo-allergic drug reactions [26].

Other than body-produced peptides, MRGPRX2 was shown to bind with diverse externally delivered agonists such as insect venom chemical components and many drugs [27]. Therefore, MRGPRX2 inhibitors are expected to be tested in MC-related medical conditions with few effective therapeutic agents, such as postoperative pain, migraine, and drug-induced acute pseudo allergic reactions [28].

### 2.2. Contribution of Mast Cells to the Pathology of the Mammalian Testis and Epididymis

**Figure 2:**
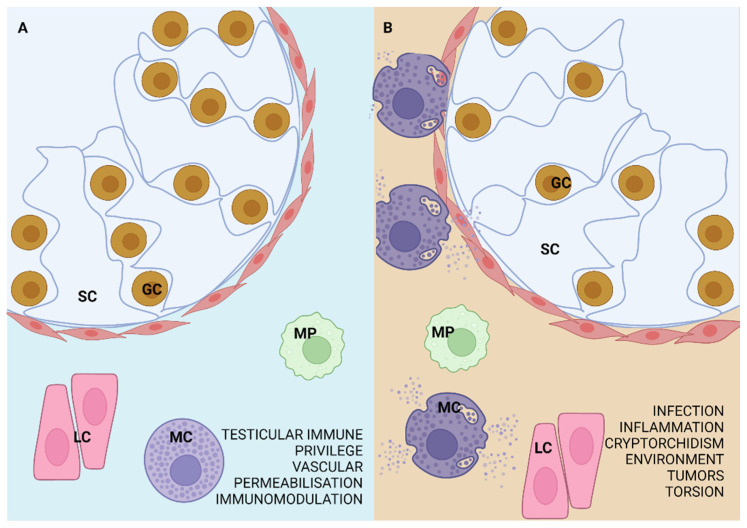
MCs in the (**A**) regular and (**B**) pathologically affected testis. The testis typically contains MCs that have a role in vascular permeabilization, testicular immune privilege, and immunomodulation. If the testis is affected by infection, inflammation, environmental factors, tumors, cryptorchidism, or testicular torsion, MCs increase in number or degranulate and may lead to the severity of the fibrosis, even germ cell loss and tubular wall thickening. SC—Sertoli cell, GC—germ cell, LC—Leydig cell, MP—macrophage.

MCs that reach and reside in the testis may be (a) regular MCs—quiescent and function physiologically or (b) pathological MCs, activated after residing in the testis or arriving de novo when related to the pathological process (Figure 2). They are typically found in the connective tissue of testis’ tunica albuginea or the epididymis in most mammals. Unlike rodents, human testes contain MCs in the interstitial tissue under physiological conditions [29,30,31]. This difference or why MCs distribute to the interstitium or just degranulate in some experimental animal models is still not elucidated. MCs contribute to the immune privilege of the testis and the homeostasis it maintains [32] by their general role in vascular permeabilization and immunomodulation, but also have suggestable roles in spermatogenesis, supported by the existence of MC—spermatozoa interaction through the binding of tryptase and proteinase-activated receptor-2 (PAR-2) [33,34].

Several pathological conditions are related to MC active response in the mammalian male reproductive system, such as infection or inflammation, testicular torsion, immunological factors, cryptorchidism, environmental factors, tumors, epididymis dysfunction, excurrent ducts obstruction, and every one of them is a possible cause of sub- or infertility [16].

For instance, testicular fibrosis, as one of the most severe infertility diagnoses, could be related to a long-term MC pro-inflammatory response usually followed by fibrogenic actions. Fibrosis occurs as the effect of extensive scarring and overgrowth after fibroblast activation into fibrotic-phenotype myofibroblasts, secreting collagen and fibronectin. MC fibrogenic activity is established through secretion of tryptase, chymase, histamine, TGF- β1, IL-13, IL-9, CCL2, PDGF, and glycosaminoglycan FGF-2 from their granules, although some, such as chymase or metalloproteinases, could have an anti-fibrotic effect, reviewed in Zhang and Kurashima [3].

The specific pathways of MC regulation in testicular pathologies remain uncharacterized. One of the reasons could be that human testicular and epididymal pathologic conditions are primarily investigated in an already developed form, which decreases the possibility to investigate the MC-caused damage mechanism or their behavior in the activation phase. Suitable animal models give more mechanistic insight into phases of disease progression.

## 3. Evolutionarily Conserved Mast Cells

Mammalian mast cells have exquisite evolutionary conservation. Some data suggest they (or their earlier forms) appeared about 450–500 million years ago in a common ancestor humans share with hagfish, lamprey, and sharks, even before adaptive immunity or chorda development [35]. The same morphology and histochemical appearance were found in the sea squirt (*Ciona intestinalis*) test cells, which already show some similarities with human mast cells, such as prostaglandin D_2_ production. The latter contain granules that store histamine and heparin-serine protease complexes. When test cells are activated, they produce prostaglandin D_2_ like MCs and are considered their counterparts in *C. intestinalis* [36].

While birds have MCs residing in the epididymis and no reported MCs in the testis [36,37,38], amphibians, with their representative, frogs (*Rana esculenta*), are a standard model of testicular MC investigation. The testes of frogs have been investigated at the light and electron microscope level and showed scarce MCs residing in the testicular interstitium, just like in reptiles (lizard, *Podarcis s. sicula* and crocodile, *Caiman crocodilus*) [39,40,41,42,43]. However, the seasonal changes during the annual reproductive cycle in testicular MC degranulation and number in the frog and lizard (a peak in early winter and late spring) are not a feature easily compared to human tissues.

## 4. Mast Cell Detection Methods in the Mammalian Testis and Epididymis

Despite the proven existence of MC in the male reproductive system, during this literature review, noticeable incoherency of methods used to locate MCs was found, including the fixation and staining method and tissue sampling (Supplementary Table S1). Most authors clearly state that the measurements, histopathological or molecular (real-time PCR, quantitative PCR, high-performance liquid chromatography (HPLC)), were carried out in whole testes (connective tissue of tunica albuginea and interstitium) [44,45,46,47,48,49,50,51], some studies analyzed MCs only in the interstitial compartment [52,53], while in a few studies it was not specifically reported [54,55,56,57]. This could lead to a decrease in the consistency of the results between studies since most MCs reside in the connective tissue of the tunica albuginea in most animal species [58].

The most considerable influence of fixation on MC detection is related to MCTs (mucosal, tryptase-only), which require fixation in non-aldehyde solutions (Carnoy) and cannot be detected with formalin fixation, like MCTCs (connective tissue, tryptase, chymase, and carboxypeptidase) can, which are not sensitive to formalin [59,60]. The previous findings are not an insurmountable problem in the testicular detection of MCs, as in the testis, almost only connective-tissue MCs are found. Parallel MC counting was performed from Bouin-Hollane’s fluid-fixed, paraffin-embedded and 2% phosphate-buffered glutaraldehyde-fixed, Epon-embedded specimens, both stained with toluidine blue dye [61] to obtain a correct measurement of possible MC total volume increase per testis, while the volume of MCs per testis may be variable due to cell number and single-cell volume. Average MC volume was different in the differently embedded sample groups. Another fixative comparison was performed regarding epididymides, fixed in either Schaffer solution (containing formalin) or BLA (basic lead acetate) to qualitatively distinguish MCs primarily found in the connective tissue or mucosa [62].

Regarding the MC tissue visualization, toluidine blue on paraffin- or resin-embedded tissues is still the most commonly used, while historically one of the oldest MC detection methods, alone or in combination with another method—Giemsa, alcian blue, safranin, aldehyde fuscin, or immunohistochemistry. Toluidine blue is a metachromatic (a pH-dependent dye that stains cell elements a different color from the dye), staining heparin-containing granules purple or red [2,63]. As a simple, non-sensitive chemical, it can be applied to tissues after various methods of fixation and embedding [44,46,47,49,50,51,53,55,57,58,61,62,64,65,66,67,68,69,70,71,72,73,74,75,76,77,78,79,80,81,82,83,84,85,86,87,88,89,90,91,92]. However, its major disadvantage is the inability to distinguish immature from mature MCs, which could be done by alcian blue-safranin staining [45,48,67,72,93]. Moreover, alcian blue-safranin can help distinguish connective tissue MCs from mucosal MCs, although it may not be necessary for the testicular MC analysis, where most, if not all, MCs are connective tissue-type [83].

Immunohistochemical markers detecting MCs in the testis include specific MC proteases (carboxypeptidase, chymase, and tryptase) [30,94], but also KIT (CD117) [95], which also stains Leydig cells, seminiferous epithelium, and the sperm acrosome [96]. One group of authors used 5-hydroxytryptamine (5-HT) or 5-HT receptor subtypes as a marker of MCs [97], analyzed by immunohistochemistry, although it has been shown that only 40% of alcian blue-positive MCs stain with 5-HT [83]. In rat tissues, an antibody against rat mast cell protease 1 (RMCP1) was used next to toluidine blue dye [49,64]. Several other, less specific markers are used in immunohistochemistry for MC detection but were not applied in testis investigations to our knowledge, such as FcεRIα (α-chain of the high-affinity IgE receptor Fc region) [97]. Further detailed summary on MC markers, in general, may be found in reviews regarding staining [12,98] and oriented on detection of MCs by flow cytometry [99,100].

## 5. Mast Cells in Mammalian Testes and Epididymides

**Figure 3:**
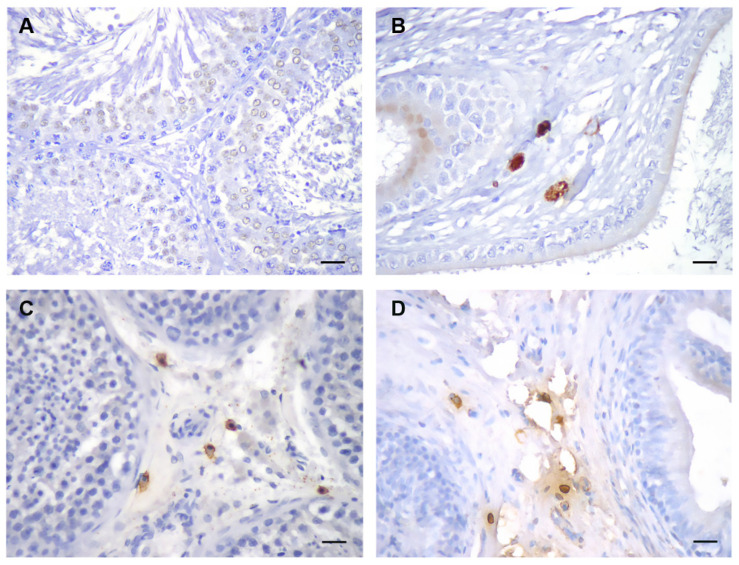
Representative images of rat testis (**A**) and epididymis (**B**), stained with an antibody against mast cell (MC) tryptase. Unlike the rat, human testicular interstitium (**C**) typically contains MCs; (**D**) human epididymis. DAB stain, hematoxylin counterstain, scale bar 25 µm.

MCs most commonly reside in the connective tissue in the testicular tunica albuginea (TA) and epididymis. In human testes, mast cells are abundant both in the subcapsular connective tissue of the TA and the interstitial tissue between the seminiferous tubules. MCs in humans appear in the testes already in the fetal period; their number increases during infancy, decreases in childhood, and again increases at the onset of puberty [29,30] (Figure 3). During development, MCs appear in the rat testes on postnatal day (PND) 30, in, or under tunica albuginea, and increase in number, especially in old age (18–24 months) [31].

### 5.1. Rodents (Rat, Mouse, Hamster, Other)

Literature data on the presence of mast cells in rat (*Rattus norvegicus*) testes are somewhat inconsistent. Mainly Wistar and Sprague-Dawley strains were used in the studies and are systematized in Supplementary Table S1. Only one study compared the results of MC analyses between rat strains [67] and showed a significant difference in the results. There is even a report showing no evidence of mast cells in the untreated rat testis [54], but without a detailed description of whether there was an occasional MC in the TA or the author implied finding no MCs in the testis proper, primarily composed of the seminiferous tubules and interstitium [101]. Due to the abundance of MCs found around subcapsular blood vessels, it has been emphasized that the number of MCs could have been under-estimated if the samples used were not whole-mounted testicular capsules alongside with testes [46].

When mentioned, data regarding MCs in the rat epididymis are consistent, repeatedly confirming MC in a noticeable number in the connective tissue around the epididymal tubules in all parts of the epididymis (head, body, and tail) [49,51,54,62,65,68,70,97].

Majeed reported finding MCs in the mouse (*Mus musculus*) epididymis but not the testis; however, as in the study analyzing rat testes, without the specification if there might be some MCs in the TA [55]. Several authors report no MCs in the regular mouse testicular interstitium [75,76,78,84], respectively analyzed in the neonatal, prepubertal, and adult [75].

Syrian (golden) hamster (*Mesocricetus auratus*) is a seasonal breeder, also having MCs positioned in the connective tissue of the TA [83,102], with only an occasional MC in the intertubular area. Under a long photoperiod (14:10 h light/dark), the MC number gradually increased from PND 23–90 (sexual maturation) and decreased during a short photoperiod (6:18 h light/dark) [83].

### 5.2. Domestic (Sus scrofa domestica) and Wild Boar (Sus scrofa ferrus)

MCs in the testis of domestic boar have a similar spatial distribution to the human testis. They inhabit both the TA and the interstitial tissue in a lower number [58,79,85,87]. Concerning the postnatal developmental phases of microminipigs, MCs appeared in the TA at birth and gradually inhabited the interstitium (interlobular area, rete testis, peritubular areas) at 1.5 months of age onward, even before they reach sexual maturity at 4.5 months [86]. No significant differences were found in the MC location and appearance (elongated and showed small cytoplasmic granules) between domestic and wild boar [58].

### 5.3. Non-Human Primates

MCs of rhesus monkey testes can be identified from the infantile period (earliest reported 100 PND), increase in number until adulthood (6–8 years), with a significant increase at peripubertal stage (3–4 years) [103]. Data obtained analyzing the common marmoset monkeys (*Callithrix jacchus*) testis show no MC markers (tryptase, chymase) detected with real-time PCR, but mention detecting MCs by immunohistochemistry [104].

References with data on MC localization in the mammalian testes or epididymides are further systematized in Supplementary Table S1, comparing MC location, fixation, detection method, and animal strain, if applicable, together with MC localizations in testes and epididymides of animals mentioned in one or a small number of studies, like the testis or epididymis in bull, deer, ram, cat, dog, hare, and some other animals.

## 6. Experimental Models Investigating Mast Cells in Mammalian Testes

The reasons behind MC presence in the normal testis are still not completely elucidated, but the disturbance of MC homeostasis is found in certain pathological conditions. In human testes, the increase in abundance of interstitial MCs is thought to lead to the disruption of spermatogenesis [105] and testicular histology [106] and consequently to male infertility [107]. The activation of inflammatory mediators and immune cells was found to precede the depletion of germ cells in many forms of infertility (e.g., cryptorchidism, Klinefelter’s syndrome).

In experiments of mast cell activation due to pathological changes, rats were the most commonly used animal models, followed by mice and, sporadically, hamsters and boars. Several of them include mechanistic data on MC degranulation or distribution. Table 1 contains the reviewed animal studies that include MCs as a primary investigation goal or a secondary finding. Physiological changes, like seasonal testicular involution or the long and short photoperiod effects, are briefly discussed and were not included in Table 1.

### 6.1. Gonadal Effects of Medications

Alkylating agents, usually administered as oncological treatment [108], cause various testicular alterations, such as germ cell loss and seminiferous tubule histology deterioration, and affect MCs [57,84,109]. The effect they have on germ cells differs based on age. If administered in an adult animal, the germ cell loss and seminiferous tubule histology deterioration are transient, but if a young, prepubertal animal receives treatment, the recovery is not possible. One example is cyclophosphamide, causing an increase in MC number and other testicular alterations that the zinc oxide nanoparticles concomitant treatment prevented [57]. The mouse model demonstrated that the changes caused by cyclophosphamide were mainly due to oxidative stress. An antioxidant, ethyl pyruvate, showed a significant reduction of the MC number elevation [84], almost to the control levels, after treatment with cyclophosphamide.

Administration of a second alkylating agent, ethylene dimethane sulphonate (EDS), which disrupts Leydig cells, has led to numerous MCs in the peritubular area in adult rat testes after Leydig cell destruction. MCs disappeared once a new Leydig cell population was differentiated [65], thus implying a novel role of MC in the induction of differentiation after histological injury. It seems that EDS did not directly affect MCs. Another proof comes from the findings that after EDS treatment of adult rats, no differences in MC number in the TA or testicular fluid were found [46]. A detailed study by Gaytan et al. elucidated the origin of MCs populating the testicular interstitium after treatment with EDS, gonadotropin-releasing hormone (GnRH) antagonist, and hypophysiotomy. Mitotic MCs exist in EDS- and GnRH antagonist-treated group testes before differentiated MCs (detected by toluidine blue and granules quality). They relate the accumulation of MCs to the local proliferation and differentiation of MC precursors [61]—blood-borne and derived from hematopoietic stem cells [110].

Moreover, inflammatory reactions may not necessarily cause the accumulation of MCs, as an inflammatory reaction would have caused the migration of other cell types (e.g., leukocytes). In contrast, GnRH antagonists and estrogen did not cause apoptosis, necrosis, or other inflammatory reactions [61]. The study has also highlighted certain relations between MCs and interstitial Leydig cells, suggesting the possibility of their common regulatory pathways. Support for this theory comes from further studies with EDS and testosterone treatment, where MC appearance in the EDS-treated testicular interstitium could also be facilitated with prolonged post-EDS testosterone administration for up to 2 months, while oxytocin treatment did not affect MC number [66].

Leydig cell destruction by EDS treatment led to a significant increase in interstitial MC number. However, the rate of MC proliferation was lower in the group additionally treated with testosterone implants (used for Leydig cell recovery), showing there are two separate phases of MC proliferation in the testes, regulated differentially. The authors suggest that mast cells are (in)directly regulated by Leydig cells [53]. Treating young newborn rats with EDS revealed a significant increase in MC number in the testes, together with their invasion in the interstitium [69].

Cl_2_MDP (dichloromethylene diphosphonate) has been used as an immuno-modulating anti-osteoclastic drug to treat hypercalcemia associated with cancer but exhibits severe macrophage cytotoxicity. As a result of macrophage depletion and consecutive inhibition of Leydig cell number increase during postnatal development after dichloromethylene diphosphonate-containing liposomes (Cl_2_MDP-lp) injection, proliferating MC number was increased in the testicular interstitium after treatment [71], again showing that Leydig cells and MCs share regulatory factors.

Some antiviral compounds also affected MC numbers, such as acyclovir, a common drug used for *Herplex simplex* virus types 1 and 2 treatment. While known to be gonadotoxic [111,112], it additionally causes an increase of peritubular and interstitial MCs in testes of adult male rats in a dose-dependent manner [50].

Recent studies relate MC activation to the environmental xenobiotics: increased MC markers Cd13, Cd33, and Cd38 in the testicular tissue in male offspring of female rats simultaneously exposed to the phytoestrogen genistein and the antiandrogenic plasticizer di-(2-ethyhexyl) phthalate during gestation [56]. These studies opened many questions and investigation possibilities on drugs affecting MC activation.

### 6.2. Mast Cell Antagonists

A limited number of studies on animal models analyzed the role of MC antagonists. In humans, common MC blockers that modulate allergic conditions include antihistamine drugs or mast cell stabilizers. Some of them are ketotifen, tranilast, fexofenadine, and ebastine; however, only the first has been analyzed in a rat model of undescended testes. The possible reason for the rarity of studies could be related to the long and common usage of MC stabilizers in human medicine, although not for infertility treatment or prevention as the main indication. Also, there is a difference in ketotifen metabolism between rats and men [113]. Ketotifen has been experimentally used in men as a treatment for oligo- and astenozoospermia and improved sperm quality and quantity [114,115,116].

Acikgoz et al. showed that in the experimental unilateral undescended testis model, a significant increase in interstitial MC number in both the descended and undescended testes was found, except with a milder change in subcapsular scrotal MC number. Ketotifen administration reduced those numbers significantly in rats of different developmental stages (prepubertal, pubertal, and adult rats) and showed a promising effect on fertility preservation [73]. Moreover, ketotifen administration reduces MC number and damage in the testicular tissue, both after autoimmune orchitis and testicular torsion (contralateral testis) [117]. Ketotifen administration after testicular damage caused by wide needle puncture also revealed its contribution to reparation and regeneration of the testis by reducing non-functioning tubule number and increasing the number of normal spermatogonia. Here, MC inactivation by ketotifen did not prevent the destructive processes of damaged testicular tissue but still significantly and positively affected the testis’ regenerative capacity [74].

Other MC antagonists were used in human studies analyzing testicular changes only and were comprehensively reviewed in Haidl et al. [16].

### 6.3. Experimental Autoimmune Orchitis (EAO)

Experimental autoimmune orchitis (EAO) study represents a combination of physical and immunological influence on the testes, causing both degranulation and an increase in the number of MCs, with their localization in the interstitium, and severe germ cell depletion, even aspermatogenesis, and interstitial damage. EAO showed a significant subcapsular and interstitial increase in MC number in rats [47,67] and mice [77]. In addition, EAO led to significant MC degranulation, and the MCs were found in the proximity to protease-activated receptor-2 (PAR_2_)-positive cells, suggesting that PAR_2_, expressed by peritubular cells, is activated by tryptase from the MCs [34,118]. In humans, spermatogonia were PAR_2_-positive cells located basally in the seminiferous epithelium [119], although, in rats, only spermatid acrosomes were PAR_2_-positive within the seminiferous tubules [47]. This could further explain the interspecies differences related to the investigation of MCs in the testis. Notably, a later study by Lustig et al. showed that these results of EAO were possibly strain-dependent: the increase in MC number (mainly in the tunica albuginea) was two-fold in Sprague-Dawley rats, and five-fold in Wistar rats 80 days after EAO, compared to the control group [67].

### 6.4. Stress

Evidence is emerging to support the role of stress in MC changes in number and maturity rather than migration. Spermatic cord torsion (with or without subsequent detorsion) injury experiments in rats showed an increase in MC number [120] although no MC migration to the interstitium in the experimental group [64], but significant MC degranulation, except in the mouse model [75]. MC antagonists or vasoactive intestinal peptide (VIP) could prevent MC degranulation caused by testicular torsion [48]. Experiments with testicular torsion on mice caused MC invasion to the interstitium of the contralateral testis postoperatively [75]. Germinal epithelium sloughing, seminiferous tubule atrophy, and interstitial edema were common in histological analyses in these experiments.

Stress caused by immobilization and low temperatures caused maturation and degranulation of MC in the testicular interstitium. In comparison, β-endorphin caused a less pronounced effect on the same specimens, while VIP significantly decreased the number of mature mast cells and inhibited degranulation [72].

### 6.5. Hormones

Experimental studies analyzing MCs concerning hormones are mostly done on rats. A series of studies by Gaytan et al. reported experimental treatments of rats on PND 1 with estrogen and findings of an increased number of MCs in the testicular interstitium and even in the lamina propria of the seminiferous tubules [44,46,70]. Notably, the same study demonstrated a maturation process of MCs in the interstitium, starting from the appearance to the fully maturated form. Recruitment of precursors and proliferation of immature MCs (recognized by mitotic figures) happens simultaneously in prepubertal MC number increase [45]. These studies showed that MCs invade the testis proper in their mature form while immature MCs proliferate and mature in the testicular interstitium after an induction signal.

Estrogen treatment on PND 1 also caused an increase in the number of MCs in whole-mounted tunica assessment. On the contrary, treatment with testosterone on PND 1 did not affect the MC number, which implies the specific role of estrogen in regulating MC proliferation in the male reproductive system [70].

### 6.6. Genetically Altered Animals

Although many experimental models show a change in MC number or localization together with germ cell depletion, only genetically altered animals may show a more specific effect that MC function has on testicular germ cells.

Several methods of genetic alterations targeting MCs are available to date. The standard models used for several decades are mice with mutations located in the *white spotting* (*W*) locus (i.e., *c-kit*), which exhibit reduced *c-kit* tyrosine kinase-dependent signaling and profound mast cell deficiency. *c-kit* mutations such as *Kit^W^/Kit^W−v^* (point mutation in the kinase domain of the receptor) [121] exhibit severe abnormalities (e.g., severe anemia and sterility) [21], while others do not (e.g., *KitW−sh/Kit/W−sh* bearing spontaneous *W-sash* (*W^sh^*) inversion mutation affecting *c-Kit* transcriptional regulatory elements) [122]. These differences could affect the conclusions on MC’s role in testicular homeostasis. For example, sterility is most probably caused directly, as KIT is also expressed in germ cells [17,123].

More recently, several strains of mice with *c-kit*-independent constitutive MC deficiency have been described for either the entire MC compartment or specific subtypes (MMC and CTMCs) [124]. In the context of fertility, *c-kit*-independent MC-deficient models have shown impairment in embryonic development. Here we discuss other genetically altered animals, where MCs have a distinct localization, number increase, or specific effect on the testis.

Transgenic male mice expressing human P450 aromatase cDNA under the control of the ubiquitin C promoter (AROM+) presented infertility as a major phenotype. They also had an increase in the number of activated mast cells in the interstitial spaces of the older mice, compared to wild-type, where no MCs were found [76], together with interstitial fibrosis.

Another model for examining postnatal Leydig cell differentiation is anti-Müllerian hormone (AMH) over-expressing mice (Mt-hAMH mice). AMH has an inhibitory effect on the regulation of postnatal Leydig cell differentiation, and MCs are activated by the consequential hormone level and spermatogenesis disruption. Their testes are deficient in Leydig cells and have many MCs in the interstitial compartment compared to controls (C57BL/6 mice) [125].

The only example of MC appearance within the seminiferous tubule, among primary spermatocytes near the basement membrane, is observed in retinoid-related orphan nuclear receptor alpha (RORα)-deficient mice, demonstrating disruption of Sertoli–germ cell junctions and showing the necessity for RORα protein in the regulation of testicular structure [78].

### 6.7. Cryptorchidism

Like the human testis, the unilateral cryptorchid testes of boar contain scarce MC in the interstitium, but their number significantly increases in the bilateral cryptorchid testes [29,87,126]. The already mentioned unilateral rat cryptorchidism model showed a mild increase in scrotal testis MC number and a high increase in the abdominal testes [127], which ketotifen, an MC antagonist, reduced [73]. No data on the effect of any MC antagonist on the pig testes were found in the available literature, although pigs have a more similar testicular MC distribution compared to humans than rats for their normal MC appearance in the interstitium.

**Table 1:** Experiments on animal models analyzing mast cells in the testis.

Animal	Strain	Experiment	Analysis	Result—MC	Result—ST	Result—Interstitium	Fertility *	Ref.
**ALKYLATING AGENTS**								
**RAT**	Wistar	EDS daily PND 5–16	PND 6–108	MC in interstitium at PND 17–35 and increased number of MCs under the TA PND 17–108	valuolae in SCs, thick basal lamina from PND 11; atrophic ST with no GCs or lumen from PND 28; no recovery after treatment	no LC from PND 11	NA	[69]
	Sprague-Dawley	EDS single dose	0–49 days after treatment	MCs in interstitium, max. 21 days after EDS	ST volume decrease: lowest 14 days after treatment; control levels 35 days after treatment	no LC on day 3 after EDS, recovered 50% by day 28 after EDS	NA	[65]
	Sprague-Dawley	1. EDS treatment ± testosterone implant 2. immunization against oxytocin	3–10 weeks after treatment	1. MCs in interstitium 21 and 70 days after EDS treatment 2. no MCs in interstitium after oxytocin administration	1. 3–4× increased GC degeneration 2. 2× increased GC degeneration	1. LC depletion	1. transient subfertility in 50%	[66]
	Sprague-Dawley	EDS ± testosterone	3–41 days after treatment	MC increase in interstitium 21–41 days post-treatment (EDS); slower increase with testosterone	NA	LC depletion, initial macrophage increase (3–10 days), then depletion, prevented by testosterone	NA	[53]
	Wistar	1. hypophysectomy ± EDS (adult) OR GnRH antagonist (prepubertal); colchicine 2. GnRH antagonist	1–30 days after treatment	1. MC proliferation on day 20 by EDS, MC number increase 15–30 day (control) and 15–50 day in hypophysectomiy + EDS 2. prolif. and diff. MC PND 23–30	NA	1. mitosis-3rd and 18–22nd day 2. immature LC and mitotic figures increase, normal by 30th day	NA	[61]
	Sprague Dawley	cyclophosphamide ± ZnO NP	after 4 weeks of treatment	MC number increase in interstitium; ZnO NP reduced (near normal MC number)	ZnO NP reduced ST and GC atrophy, separation of germinal epithelium from BM, tubular wall vacuolization, sperm abnormalities	wide interstitium, vascular congestion, acidophilic material, clusters of dark LC; ZnO NP reduced	NA	[57]
**MOUSE**	NMRI	cyclophosphamide ± EP for 35 days	after treatment	MC number increase in interstitium; EP reduced	germinal epithelium disarrangement and thickening; EP reduces	LC depletion; EP reduced	NA	[84]
**INFLAMMATION**								
**RAT**	Wistar and Sprague-Dawley	EAO (3 injections every 15 days)	7–80 days after 1st injection	MC degranulation, number elevation after 80 days, 2× Sprague-Dawley and 5× Wistar	aspermatogenesis at day 80	mononuclear cell infiltrate at day 80	NA	[67]
	Wistar	EAO	50 & 80 days after EAO	MC number and degranulation increase; MC in interstitium	almost complete loss of GC	granuloma formation; PAR_2_+ cell number increase	NA	[47]
	Wistar	testis puncture Ø 3 mm (=EAO) followed by skin suture ± ketotifen	1–30 days after treatment	ketotifen reduced MC number and degranulation increase in interstitium	GC depletion and ST fibrosis; ketotifen (does not prevent damage but) retains regenerative capacity		NA	[74]
	Wistar	EAO ± ketotifen	56 days after first treatment	ketotifen reduced MC number increase	ketotifen reduced severity of histopathological testicular damage	ketotifen reduced testicular damage	NA	[117]
**MOUSE**	C57BL6/N or C57BL/6J	EAEO	30–80 days after 1st immunisation	MC number increase in interstitium from low to high-grade EAEO	severe EAEO: ST diameter reduction, germ cell sloughing, SC-only tubules	macrophage and leukocyte number increase, fibrosis around ST	NA	[77]
**TORSION AND STRESS**								
**RAT**	Sprague–Dawley	torsion ± ketotifen	30 days after treatment	ketotifen reduced increased MC numbers—contralateral testis	ketotifen reduced germinal epithelium sloughing, ST atrophy, walls fibrosis—contralateral testis	interstitial edema	NA	[117]
	Sprague-Dawley	torsion-detorsion ± VIP (2 or 4 h); prepubertal	after torsion	MC degranulation; VIP—protective effect (2 h); no MCs in interstitium	histological abnormalities; VIP reduced (2 h torsion)	histological abnormalities; VIP reduced (2 h torsion)	NA	[48]
	Sprague-Dawley	torsion; contralateral testis analysis	10, 30, and 80 days after torsion	maximum increase in MC number 30 days post-op	germinal epithelium sloughing (spermatocyte and spermatid apoptosis), ST atrophy, fibrosis, diameter, and Johnsen score decrease; possible reversibility	interstitial edema, T-lymphocyte, and macrophage number elevation	NA	[64]
**MOUSE**	dYY	torsion; contralateral testis analysis	4–24 weeks after torsion	MC number increase in interstitium, maximum 8 weeks post-op	no histological changes	no histological changes	NA	[75]
**RAT**	Wistar	1. immobilisation cold stress 3h 3 days PND 15, 30, 45 ± VIP 2. β-endorphin + immobilisation PND 45 ± VIP	after treatment	1. VIP reduced MC degranulation and maturation 2. VIP reduced MC degranulation and maturation	NA	1. VIP prevented focal LC depletion	NA	[72]
	Wistar	torsion/detorsion ± hypothermia for 30 or 90′ prior to detorsion	8 weeks after operation	torsion/detorsion significantly increased MC number	hypothermia increased Johansen score, reduced by torsion/detorsion	hypothermia ameliorated interstitial edema	NA	[120]
**HORMONE TREATMENT**								
**RAT**	Wistar	EB on PND 1	PND 45 and 90	strong increase of MC number, interstitium at PND 45	maturation arrest at the level of pachytene spermatocytes	increased proportion of interstitium (fibrosis and edema)	NA	[44]
	Wistar	1. EB on PND 1 2. testosterone propionate on PND 1	PND 45	1. MC number increase after estrogen in the testis 2. no effect	1. impaired spermatogenesis	1. immature interstitial cells	NA	[70]
	Wistar	EB on PND 1	PND 15–90	MC in interstitium on PND 17, increase in number by PND 45 and mature by PND 90	NA	mature LC from PND 90	NA	[45]
	Wistar	1. EB treatment on PND 1 2. EDS (adult)	1. PND 35–70 2. 5 days after treatment	1. strong increase of MC number, interstitium at PND 35–70 testicular serotonin increase 2. no effect on MCs	NA	1. LC depletion on PND 35; normal number on PND 70 2. LC absent	NA	[46]
**ETHANOL**								
**RAT**	Wistar	ethanol and ethanol extract of Bauhinia forficata	31 days after 1st treatment	decreased only the number of degranulated MCs	no effect	NA	NA	[68]
	UchB (Wistar)	ethanol 100 days	after treatment	no difference in MC number (testis); increase in total number of degranulated MCs (epidydimis)	NA	NA	NA	[49]
	Wistar	ethanol 54 days	after treatment	no difference (testis); increase of MC number and degranulation (cauda & initial segment)	ethanol: mature spermatid number, mobile sperm count reduction; abnormal seminiferous tubule morphology	NA	NA	[51]
**GENETIC ALTERATIONS**								
**MOUSE**	WT and transgenic AROM+	AROM+ alteration	4-, 9-, and 15- month-old	MC number increase in interstitium and during aging	spermatogenic disruption progression during aging (GC depletion to absence at 15 months)	LC hyperplasia and hypertrophy (4 months), giant multinucleated macrophage number progression; LC adenomas (9- and 15-month-old)	NA	[76]
	Mt-hAMH and C57BL/6	Mt-hAMH alteration	5-month-old	abundant MC in the interstitium	lower length of ST, vacuolization of Sertoli cells, loss of GC	LC depletion	infertility after 3 rounds of consecutive pairing	[125]
	WT (C57BL/6) and RORα-deficient	RORα-deficiency alteration	10–12-week-old	MC in the interstitium and within the seminiferous tubule near BM	ST diameter and germinal epithelium height decrease (GC apoptosis), basal membrane irregularities, hypospermatogenesis	LC vacuolization number reduction	NA	[78]
**INFECTION**								
**RAT**	Wistar	*C. trahomatis* inoculation	3–90 days after infection	MCs in inflammatory lesions of the epididymis 30 days after	germinal epithelium loss, spermatid giant cells after 7–70 days	interstitial fibrosis after 7–70 days	NA	[128]
**DEER MOUSE**		*Trypanosoma brucei* infection	0–10 weeks after infection	MC number increase in the interstitium	ST diameter decrease (loss of GC), increase with time	LC accumulation, mononuclear cell infiltration, increase with time	NA	[129]
**CRYPTORCHIDISM**								
**RAT**	Wistar	experimental UDT (newborn) ± ketotifen, then peripubertal, pubertal, or adult bilateral orchidectomy	after orchidectomy	experimental UDT increased, and ketotifen decreased MC number in scrotal and abdominal testis	UDT: ST diameters decreased, ST basement membranes thickened, and spermatogenesis decreased—both testes; ketotifen prevented	interstitial fibrosis; ketotifen prevented	NA	[73]
	Sprague-Dawley	PND 15–17 unilateral cryptorchidism	15 days after treatment	MC number increase in cryptorchid (higher) and scrotal (lower) testis	ST atrophy, diameter reduction, degenerative changes, GC disconnection from BM, BM thickening, destruction in tight junctions between SCs, SCs, and spermatogenic cells, decomposition of cytoplasmic bridges between spermatogenic cells	perivascular and interstitial fibrosis, edema, congestion, hemorrhage	NA	[127]
**BOAR**	domestic	cryptorchidism (unilateral and bilateral)	9-month-old	abundant MCs in bilateral cryptorchid testes interstitium	NA	unilateral: fibrosis and LC degeneration in abdominal testis bilateral: advanced fibrosis, immature LC and LC degeneration	NA	[87]
**OTHER**								
**RAT**	Sprague-Dawley	topical histamine, ritanserin, ketanserin, histamine, and substance 48/80	30 min after treatment	MC degranulation after histamine and substance 48/80 (dose-dependant)	NA	NA	NA	[92]
	Wistar	acyclovir i.p. 15 consecutive days (3 doses)	18 days after last treatment	increase of MC number in the testicular interstitium and peritubular area with higher doses	ST diameter, epithelial height (cell loss), tubular differentiation index, spermiogenesis index, repopulation index (higher dose) decrease	LC atrophy, connective tissue increase	lower pregnancy rate	[50]
	Wistar	1. Cl_2_MDP-lp injection PND 5, 10, 15, 20, 25 2. Cl_2_MDP ± hCG & hFSH PND 18 for 6 days	1. PND 10–35 2. PND 27	1. MC in interstitium 10 and 15 days after treatment	NA	1. short-term LC depletion after treatment PND 5–15 and long term LC depletion after treatment on PND 20–25 2. LC depletion, no changes after hCG and hFSH treatment	NA	[71]
	Wistar	males with no mating experience and colony breeders analyzed at PND 40–170	PND 40, 60, 90, 120	serotonin + MC increase PND 40- 90; MC number at peak on PND 90	NA	NA	NA	[97]
	Sprague-Dawley	genistein ± DEHP in utero	PND 60, 120, 180	genistein + DEHP: MC marker increase on PND 120	genistein + DEHP: disruption in Sertoli cell function, different stage spermatogonia change	DEHP: LC number decrease PND 120	NA	[56]

* fertility analysis by the pairing of males after treatment. MC—mast cell, ST—seminiferous tubule, EDS—ethylene diethylstilboestrol, PNDpostnatal day, GCgerm cell, TA—tunica albuginea, LC—Leydig cell, NA—not analyzed, SC—Sertoli cell, GnRH—gonadotropin-releasing hormone, ZnO NP—zinc oxide nanoparticles, EP—ethyl pyruvate, EA(E)O—experimental autoimmune (epididymo)orchitis, PAR2—protease-activated receptor-2, VIP—vasoactive intestinal peptide, EB—estradiol benzoate, AROM—mice expressing human P450 aromatase cDNA, Mt-hAMH—AMH over-expressing mice, RORα—retinoid-related orphan nuclear receptor alpha, BM—basal membrane, UDT—unilateral descendent testis, Cl2MDP—dichloromethylene diphosphonate, hCG—human chorionic gonadotropin, hFSH—follicle-stimulating hormone DEHP—di-(2-ethylhexyl) phthalate. If not stated otherwise, adult, sexually mature animals were used. Underlined are substances that reduced or ameliorated the experimental effect on MCs.

## 7. Experimental Models Investigating Mast Cells in Mammalian Epididymides

Ethanol. Experimental observations of MCs in the epididymis mainly include ethanol intake. Ethanol-preferring rats showed an increase in the total number of degranulated MCs in the epididymis, but no such effect was observed in the testis [49]. In prepubertal rats, an increase in MC number and degranulation in the caudal and initial segment of the epididymis after ethanol consumption was observed [51]. Antioxidants seem to have a protective effect in such experiments. Alternating intake of an antioxidant *Bauchinia forficata* alcoholic extract compared to only ethanol intake for 15 days relieved the MC degranulation level in the epididymal head [68].

Hormones. Contrary to the testis, neonatal estrogenisation did not cause significant MC number change in the rat epididymis; instead, it was related to the increased volume. Similarly, testosterone administration on PND 1 did not affect epididymal MCs in the prepubertal testis [70].

Inflammation. Inoculating *C. trahomatis* to the vas deferens caused pyogranulomatous inflammation, abscesses, and spermatic granulomas in the rat epididymis. MCs are typical in moderate to severe interstitial inflammation, next to lymphocytes, plasma cells, and neutrophils [128]

## 8. Discussion

Although significant progress is achieved in studies about MCs’ role in male infertility, unknown elements in the cascade of mediators in the complex pathophysiology of male infertility, which MCs significantly influence, call for further detailed studies in real-time conditions. There is limited access to human testicular tissues prior to histologically recognizable infertility. Hence, there is a great interest in finding an animal or in vitro model, which could be used in experiments analyzing the impact of various stimuli (chemical, biological, physical) on MC activation.

Available data regarding MCs in the testis abounds exquisite reviews on humans [16,33,130] and a comprehensive review on MCs in the nonmammalian vertebrates [42], including the presence of MCs in the testes of birds, frogs, and lizards. A comprehensive review on the mammalian testicular and epididymal MC has not been found written from the perspective of method and result comparison in the available literature and may have a significant impact in drawing the attention of future authors to crucial problems in MC analysis.

The evolutionary conservation provides the possibility to use the same detection method for MC analysis in the reproductive system of several mammalian species. The functions and granules contents are almost identical, while different histological features in the testicular architecture between mammals (e.g., seminiferous wall thickness) and other biological differences, such as the subtle blood-testis barrier variations between the species [131,132,133,134], could be some of the causes of variable testicular MC effect on testicular structures between humans and other vertebrates.

Molecular mechanisms of MC activation in infertility may be analyzed by several methods used generally in MC investigations. On the other hand, male infertility studies require histological assessment due to the characteristics of MC distribution and migration within the testicular tissue. As suggested by Mayerhofer et al. in 2018. and Haidl et al. in 2011, the anatomical proximity of the MCs to the testicular structures, especially seminiferous tubules, are significant in the pathology of human infertility. Logically, the closer the MCs are to germ cells, the more direct an effect they can have on fertility via secreted mediators. Due to these characteristics, it is necessary to consider the tubular wall thickness and conditions of the blood-testis barrier when discussing and analyzing the effect of MC on germ cells and fertility.

Among the animals used in MC research, domestic boars seem to have MC distribution more similar to the human testis than rodents [58,79,86,87]. If used in experiments, the breed [135] and exposure to light should be taken into consideration [136]. Wild boars are indeed not a proposed model in MC investigation, for both impractical sample collection and seasonality in testis function [137], whereas the effect of seasons on fertility became evolutionarily ameliorated in domestic pigs. They do not show such distinct changes in sperm production and quality, especially if the light exposure resembles the conditions of increasing photoperiods [136].

Hamsters, as typical seasonal breeders, may not be the most suitable model animals for MC investigation—the apoptotic and proliferative activity and the testicular involution are not features easily translated on human testicular investigations [83,102]. With respect to the difference in animal facility conditions and practicality, pigs are optimal as an animal model in testing chemicals that could aid human infertility.

Although human MCs have been divided into predominantly tryptase (MCT), chymase (MCC) or both (MCTC) and rodent MCs into connective (CTMC) or mucosal tissue MCs (MMC), with a high level of similarities between human and rodent MCs, it has been noted that perhaps an organ-specific classification should take place [138]. The previously mentioned review does not include specific MC markers in testicular tissues, but there may be subtle differences in testicular and epididymal MC expressed markers compared to MCs residing in other organs, and not just the known differences between mucosal or connective tissue MCs.

Pre-detection methods are crucial in (immuno)histochemical MC analysis, for tissue sampling, fixation, and staining may significantly alter the results. Whenever possible, whole testes should be fixated (including the tunica albuginea) while several fixations and staining methods should be tested, at least at the beginning of the study, to evaluate the most accurate method in the data collection. Toluidine blue should be included in the analyses, being the most used detection method, for better result comparison, and MC tryptase shows a limited expression in rodents [73].

Detection methods used in MC analyses also need systematization and guidelines regarding testicular studies. In general, studies on MCs in the testes still do not include a high diversity in detection methods and possible markers (Supplementary Table S1). For example, flow cytometry analysis of testicular MCs has not been performed to the best of our knowledge. In most cases, a histological assessment was used with a limited number of immunohistochemical markers. Nonetheless, any variation of histological analysis gives valuable data on MC interactions with other testicular cells (such as a change in location, degranulation, or shape), and other methods (real-time PCR, quantitative PCR, fluorescence-activated cell sorting (FACS)) cannot obtain that. A phenomenon called “phantom mast cells” occurs after extensive degranulation of MCs, which remain present but undetectable by toluidine or any other staining that detects granules [139]. In order to avoid falsely-negative results for this reason, especially in experiments analyzing MC degranulation, other detection methods should be used, like antibodies against MC tryptase, that will detect residual protease or KIT (CD 117) that does not bind to granules of MCs [140].

Regarding the in vitro model, other testicular cell types, especially Leydig cells, need to be involved in the in vitro investigations, as shown in many studies, where MCs and Leydig cells directly affect one another and share common regulatory factors [45,66,71].

Despite the enormous achievements in in vitro testicular models [141,142], MCs are still not included in the 2D or 3D testicular in vitro models. Nonetheless, significant progress has been made, for example, a study from 2020 showed a 3D co-culture model including Sertoli, Leydig, endothelial, myoid cells, and macrophages, detected by their respective specific markers [143].

There are additional obstacles to overcome prior to including MCs in one of the 3D model variations [141]. However, in the abundance of investigation on animal models and a relative scarcity of human material, the 3D models are the promising future in clarifying the MC role in male infertility. Studies including MCs should follow some general guidelines because of the specific localization, interspecies differences, and activity in the testicular and epididymal pathology.

## 9. Conclusions

- Histological assessment, including toluidine blue stain, should always be included in studies analyzing testicular and epididymal mast cells, as a standard method that stains all mast cell subtypes, regardless of protein content.
- Depending on the effect and antibody used, a few fixation methods should be optimized at the beginning of the study due to the mast cell subtype specificity.
- When investigating animal models, whole testes should be used, including the tunica albuginea, for in most mammals, mast cells reside right underneath. When found in the interstitium, the seminiferous tubule wall thickness should be commented upon.
- With respect to practicality, domestic boars kept under non-variable conditions are proposed to resemble human testicular mast cell distribution better than rodents.
- No exclusively testis- or epididymis-specific mast cell markers have been found yet, although the characterization of other organ-specific mast cell markers is known.
- 3D in vitro models are promising, although they still need significant development in order to incorporate mast cells and the tunica albuginea, if possible. Further efforts need to be made to develop a suitable human-origin testicular cell line combination.

## 10. Materials and Methods

### 10.1. Samples

Both adult Wistar strain rat (3-month-old) and human (25-year-old) samples were obtained from archive collections. Normal, disease-free samples were chosen for both specimens. Serial sections (4 µm) were cut for immunohistochemistry on a Leica microtome.

### 10.2. Immunohistochemistry

Antibody against tryptase (1:100, sc-59587, Santa Cruz Biotechnology, Santa Cruz, CA, USA) was incubated overnight at 4 °C. The next day, sections were incubated with a secondary antibody and stained with 3,3′-diaminobenzidine-tetrahydrochloride (DAB). Hematoxylin was used for counterstaining.

## Data Availability

Not applicable.

## Supplementary Materials

The following supporting information can be downloaded at: https://www.mdpi.com/article/10.3390/ijms23052547/s1.
