# Supplementary material for: Mast Cells in the Mammalian Testis and Epididymis—Animal Models and Detection Methods
Source: Int J Mol Sci. 2022 Feb 25;23(5):2547. doi: 10.3390/ijms23052547 (PMC8909951; doi:10.3390/ijms23052547)
Supplement: Supplementary file 1 [file ijms-23-02547-s001.zip › ijms-1565095-supplementary.pdf]

International Journal of
Molecular Sciences

MDPI

Supplementary Table S1. Mast cell detection, fixation, and embedding method in different mammals**.**

Animal	Strain	MC localization in the testis	MC localization in the epididymis	Fixation/embedding method	Detection method	Ref.
**Rat**	NA	TA	NA	NM	alcian blue, safranin O, berberine sulphate	[1]
	Lewis	NA	caput and cauda	Schaffer solution or BLA/paraffin	toluidine blue	[2]
	Sprague - Dawley	NM	NA	2.5% phosphate-buffered glutaraldehyde/epoxy resin	toluidine blue	[3]
	Sprague - Dawley	TA	NA	Bouin's fixative/paraffin	alcian blue/safranin	[4]
	Sprague - Dawley	TA	NA	Bouin's fixative/paraffin	toluidine blue or alcian blue, RMCP1 and MC tryptase antibody	[5]
	Sprague - Dawley	TA	NA	2% phosphate buffered glutaraldehyde/Araldite-Epon	toluidine blue	[6]
	Sprague - Dawley	NM	NA	buffered glutaraldehyde/NM	toluidine blue	[7]
	Sprague - Dawley	TA	NA	4% formaldehyde, 3% glutaraldehyde, 0.05% picric acid in 0.1 M cacodylate buffer/Epon	toluidine blue	[8]
	Sprague - Dawley	NA	NA	2% paraformaldehyde, 75 mM lysine, 10 mM periodate, containing 0.5% glutaraldehyde/OCT	toluidine blue	[9]
	Sprague - Dawley	TA	NA	10% formaldehyde, NM/paraffin, NM(EM)	H&E, Masson's trichrome stain, MC tryptase antibody	[10]
	Sprague - Dawley	NA	NA	-	RT-PCR and qPCR – Cd13, Cd33, Cd38	[11]

	UchB (Wistar)	TA	stroma, close to the epithelium, around BV, capsule connective tissue	4% paraformaldehyde and sodium cacodylate buffer 0.1 m/Paraplast	toluidine blue; RMCP1 antibody	[12]
	Wistar and CD	not found	present	NM	NM	[13]
	Wistar or Sprague - Dawley	TA	NM	Bouin's fixative/paraffin	alcian blue	[14]
	Wistar and Sprague - Dawley	TA	NA	NM/cryostat or paraffin	toluidine blue, alcian blue, safranin and alcian blue/safranin	[15]
	Wistar	TA	NA	Bouin-Hollande's fluid/paraffin	toluidine blue, alcian blue	[16]
	Wistar	NA	NM	formalin/paraffin	H&E	[17]
	Wistar	TA	NA	isopropanol/LR white resin	toluidine blue	[18]
	Wistar	NA	connective tissue (head and tail)	methacarn/paraplast	toluidine blue	[19]
	Wistar	TA	NA	frozen or 2.5% glutaraldehyde/JB4 resin	toluidine blue	[20]
	Wistar	NM	NA	Bouin-Hollande's fluid or 2% phosphate buffered glutaraldehyde/paraffin or Epon	toluidine blue	[21]
	Wistar	TA	NA	Bouin-Hollande's fluid or 2% phosphate buffered glutaraldehyde/paraffin or epoxy resin	aldehyde fuscin and toluidine blue	[22]
	Wistar	TA	found in the stroma	Bouin's fixative and 2% phosphate buffered glutaraldehyde/paraffin and Araldite	toluidine blue	[23]
	Wistar	TA	NA	Bouin-Hollande's fluid or 2% phosphate buffered	alcian blue - safranin	[24]

				glutaraldehyde/paraffin or epoxy resin		
	Wistar	TA	NA	Bouin-Hollande's fluid/NM and whole-mounted	toluidine blue and 5HT by HPLC	[25]
	Wistar	NM	NA	Bouin-Hollande's fluid/paraffin	toluidine blue	[26]
	Wistar	NM	NA	10% neutral formalin or 2.5% phosphate-buffered glutaraldehyde/paraffin or Araldite	toluidine blue and alcian blue-safranin	[27]
	Wistar	NM	NA	10% neutral buffered formalin/paraffin	toluidine blue	[28]
	Wistar	TA	stroma	methacarn/Paraplast	toluidine blue	[29]
	Wistar	NA	caput	frozen in 2-methyl butane + paraformaldehyde (4%)	HPLC, IHC (serotonin transporter & 5HT1B)	[30]
	Wistar	NM	NA	10% buffered formalin/paraffin	toluidine blue	[31]
	Wistar	NM	NA	10% formaldehyde/wax	toluidine blue and azure II	[32]
	Wistar	NM	NA	Bouin’s fixative/paraffin	H&E	[33]
**Mouse**	dYY	no MCs in interstitium	present, unspecified	NM	toluidine blue	[34]
	NA	not found	present, unspecified	NM	NM	[35]
	WT and transgenic AROM+	no MCs in interstitium	NM	4 % paraformaldehyde/paraffin	Giemsa or toluidine blue	[36]
	C57BL6/N or C57BL/6J	no MCs in interstitium	NA	Bouin’s fixative/paraffin	toluidine blue	[37]
	Mt-hAMH and C57BL/6	no MC in interstitium	NA	5% glutaraldehyde solution/Epon Araldite	Methylene Blue Azure II	[38]
	WT (C57BL/6) and RORα-deficient	no MC in interstitium	NA	3% paraformaldehyde–glutaraldehyde/Araldite	toluidine blue and c-kit antibody	[39]
	NMRI	TA	NA	10% formal saline/paraffin	toluidine blue	[40]
**Golden hamster**		TA	NA	Bouin’s fixative or 10% neutral formalin/paraffin	toluidine blue, Alcian blue, anti-5-HT serum	[41]

		TA	NA	Bouin's fixative or 5% glutaraldehyde/paraffin or Araldite	aldehyde-fuchsin and alcian blue	[42]
**Deer mouse**	Peromyscus maniculatus	no MC in interstitium	present	Karnovsky's fixative	uranyl acetate and lead citrate	[43]
**Black-footed pygmy rice rat**	Oligoryzomys nigripes	NA	interduct compartment	Karnovsky fixative/2-hydroxyethyl methacrylate	toluidine blue	[44]
**Hare**		not found	not found	Bouin-Hollande's fluid/paraffin	toluidine blue, alcian blue	[16]
**Non-human primate**	Rhesus monkey (*Macaca mulatta*)	interstitium	NA	Bouin's fixative/paraffin	MC tryptase antibody	[45]
	Common marmoset monkeys (*Callithrix jacchus*)	NM	NA	-	RT-PCR tryptase (TPSG1) and chymase (CMA1) primers	[46]
**Bat**	*Desmodus rotundus*	interstitium	NA	Karnovsky fixative/Historesin	toluidine blue	[47]
	Artibeus lituratus	NA	not found	Karnovsky fixative/Historesin	toluidine blue	[48]
**Cat**	NM	TA	NA	Bouin-Hollande's fluid/paraffin	toluidine blue, alcian blue	[16]
**Dog**	NM	TA	NA	Bouin-Hollande's fluid/paraffin	toluidine blue, alcian blue	[16]
**Pig**	domestic	TA and interstitium	NA	10% neutral buffered formalin and Bouin's fixative/paraffin	toluidine blue	[49]
	Duroc-Jersey	TA	NA	Bouin's fixative or 3.5% glutaraldehyde with 1% osmium tetroxide/paraffin or Araldite	Cajal-GaUego's trichrome method, toluidine blue	[50]
	microminipigs	TA and interstitium	NA	modified Davidson's solution/paraffin	toluidine blue	[51]
	domestic	interstitium	NA	Karnovsky's fixative with 1% osmium tetroxide / Spurr resin	toluidine blue, uranyl acetate, and lead citrate	[52]
	domestic (*S. scrofa domestica*)	interstitium	NA	Bouin-Hollande's fluid/paraffin	toluidine blue, alcian blue	[16]

	and wild (*S. scrofa ferrus*)					
**Bull**	Deutsches Fleckvieh	interstitium	NA	solutions I and II of Forssmann or Karnovsky's fixative/Epon, Araldite, ERL 4206 or Epon-Araldite	azur II - methylene blue or uranyl acetate and lead citrate	[53]
	Hereford	NA	intertubular connective tissue - head	5% glutaraldehyde/ Epon	toluidine blue or uranyl acetate, and lead citrate	[54]
	Deutsches Fleckvieh	not found	NA	Bouin's fixative or methanol-glacial acetic acids (2:1) or 3.7 % formalin solution/paraffin	toluidine and alcian blue	[55]
	Deutsches Fleckvieh	NA	intertubular connective tissue	Bouin's fixative or methanol-glacial acetic acids (2:1) or 3.7 % formalin solution/paraplast blocks	toluidine and alcian blue	[56]
	NM	TA and interstitium	NA	Bouin-Hollande's fluid/paraffin	toluidine blue, alcian blue	[16]
**Deer**	NM	TA	abundant	Bouin-Hollande's fluid/paraffin	toluidine blue, alcian blue	[16]
**Ram**	NM	NA	connective tissue, tail	10% neutral buffered formalin and Bouin's fixative/paraffin	toluidine blue	[57]
**Horse**	NM	TA and interstitium	NA	Bouin-Hollande's fluid/paraffin	toluidine blue, alcian blue	[16]
**Mule**	NM	interstitium	NA	Bouin-Hollande's fluid/paraffin	toluidine blue, alcian blue	[16]
**Donkey**	NM	interstitium	NA	Bouin-Hollande's fluid/paraffin	toluidine blue, alcian blue	[16]

MC – mast cell, NA – not analyzed, NM – not mentioned, TA – tunica albuginea, BLA – basic lead acetate, RMCP1 – rat MC protease 1, RT-PCR – real-time PCR, qPCR – quantitative PCR, BV – blood vessel, OCT - optimal cutting temperature compound, 5-HT- 5-hydroxytryptamine, H&E – hematoxylin and eosin, 5HT - 5-hydroxytryptamine, HPLC - high-performance liquid chromatography, IHC - immunohistochemistry, 5HT1B - 5-hydroxytryptamine receptor 1B, TPSG1 - tryptase gamma 1, CMA1 – chymase 1.

Reference

1. Zhou, Z., S. Shi, and S. Wang, *The development and chronological changes of mast cell in testes of rats.* Chinese Journal of Histochemistry and Cytochemistry, 1998. **7**(1): p. 26-30.
2. Fritz, F.J. and R. Pabst, *Numbers and heterogeneity of mast cells in the male genital tract of the rat.* International archives of allergy and applied immunology, 1989. **88**(3): p. 360-362.

3. Anan, H.H., et al., *Ameliorative effect of zinc oxide nanoparticles on cyclophosphamide induced testicular injury in adult rat.* Tissue and Cell, 2018. **54**: p. 80-93.
4. Can, C., et al., *Protective effect of vasoactive intestinal peptide on testicular torsion-detorsion injury: association with heparin-containing mast cells.* Urology, 2004. **63**(1): p. 195-200.
5. Rodriguez, M.G., et al., *Immunohistopathology of the contralateral testis of rats undergoing experimental torsion of the spermatic cord.* Asian Journal of Andrology, 2006. **8**(5): p. 576-583.
6. Jackson, A.E., et al., *The Effects of Ethylene Dimethane Suiphonate (EDS) on Rat Leydig Cells: Evidence to Support a Connective Tissue Origin of Leydig Cells1.* Biology of Reproduction, 1986. **35**(2): p. 425-437.
7. SHARPE, R.M., H.M. FRASER, and W.D. RATNASOORIYA, *Assessment of the role of Leydig cell products other than testosterone in spermatogenesis and fertility in adult rats.* International Journal of Andrology, 1988. **11**(6): p. 507-523.
8. Collin, O., J.E. Damber, and A. Bergh, *5-Hydroxytryptamine--a local regulator of testicular blood flow and vasomotion in rats.* J Reprod Fertil, 1996. **106**(1): p. 17-22.
9. Wang, J., et al., *Leukocyte Populations of the Adult Rat Testis Following Removal of the Leydig Cells by Treatment With Ethane Dimethane Sulfonate and Subcutaneous Testosterone Implants1.* Biology of Reproduction, 1994. **51**(3): p. 551-561.
10. Bostancierİ, N., İ. Duran, and M. Yüncü, *Investigation of Testicular Mast Cells and Fibrosis in Rats With Experimental Unilateral Cryptorchidism.* Bozok Tıp Dergisi, 2021. **11**(1): p. 7.
11. Jones, S., et al., *Disruption of Rat Testis Development Following Combined In Utero Exposure to the Phytoestrogen Genistein and Antiandrogenic Plasticizer Di-(2-Ethylhexyl) Phthalate1.* Biology of Reproduction, 2014. **91**(3).
12. Mendes, L.O., et al., *Mast Cells and Ethanol Consumption: Interactions in the Prostate, Epididymis and Testis of UChB Rats.* American Journal of Reproductive Immunology, 2011. **66**(3): p. 170-178.
13. Majeed, S.K., *Mast cell distribution in rats.* Arzneimittelforschung, 1994. **44**(3): p. 370-4.
14. Moreno, D., et al., *Effect of ketotifen fumarate on experimental autoimmune orchitis and torsion of the spermatic cord.* Asian J Androl, 2020. **22**(1): p. 112-117.
15. Lustig, L., et al., *Testicular mast cells in autoimmune orchitis.* Am J Reprod Immunol, 1995. **33**: p. 1.
16. Anton, F., et al., *A comparative study of mast cells and eosinophil leukocytes in the mammalian testis.* Zentralbl Veterinarmed A, 1998. **45**(4): p. 209-18.
17. Jantos, C., et al., *Experimental epididymitis due to Chlamydia trachomatis in rats.* Infection and immunity, 1992. **60 6**: p. 2324-8.
18. Iosub, R., et al., *Development of testicular inflammation in the rat involves activation of proteinase-activated receptor-2.* J Pathol, 2006. **208**(5): p. 686-98.
19. Sampaio, C.F., et al., *Alcohol extract of Bauhinia forficata link reduces lipid peroxidation in the testis and epididymis of adult Wistar rats.* Microsc Res Tech, 2019. **82**(4): p. 345-351.
20. Zaidi, A., et al., *Abnormal development of the testis after administration of the Leydig cell cytotoxic ethylene-1,2-dimethanesulphonate to the immature rat.* Journal of reproduction and fertility, 1988. **82**(1): p. 381-392.
21. GAYTAN, F., et al., *Simultaneous Proliferation and Differentiation of Mast Cells and Leydig Cells in the Rat Testis.* Journal of Andrology, 1992. **13**(5): p. 387-397.
22. Gaytan, F., et al., *Increased number of mast cells in the testis of neonatally estrogenized rats.* Arch Androl, 1986. **16**(3): p. 175-82.
23. Gaytan, F., et al., *Mast cells in the testis, epididymis and accessory glands of the rat: effects of neonatal steroid treatment.* J Androl, 1989. **10**(5): p. 351-8.
24. Gaytan, F., et al., *Differentiation of mast cells during postnatal development of neonatally estrogen-treated rats.* Cell Tissue Res, 1990. **259**(1): p. 25-31.
25. Aguilar, R., et al., *Testicular serotonin is related to mast cells but not to Leydig cells in the rat.* J Endocrinol, 1995. **146**(1): p. 15-21.

26. Gaytán, F., et al., *Requirement for testicular macrophages in Leydig cell proliferation and differentiation during prepubertal development in rats.* Journal of reproduction and fertility, 1994. **102 2**: p. 393-9.
27. Tunçel, N., et al., *The effect of vasoactive intestinal peptide (VIP) on mast cell invasion/degranulation in testicular interstitium of immobilized + cold stressed and beta-endorphin-treated rats.* Peptides, 1996. **17**(5): p. 817-24.
28. Movahed, E., et al., *Toxic effect of acyclovir on testicular tissue in rats.* Iranian journal of reproductive medicine, 2013. **11**(2): p. 111-118.
29. Paula Franco Punhagui, A., et al., *Ethanol exposure during peripubertal period increases the mast cell number and impairs meiotic and spermatic parameters in adult male rats.* Microscopy Research and Technique, 2016. **79**(6): p. 541-549.
30. Jiménez-Trejo, F., et al., *Serotonin Concentration, Synthesis, Cell Origin, and Targets in the Rat Caput Epididymis During Sexual Maturation and Variations Associated With Adult Mating Status: Morphological and Biochemical Studies.* Journal of Andrology, 2007. **28**(1): p. 136-149.
31. Acikgoz, A., et al., *The role of ketotifen in the prevention of testicular damage in rats with experimental unilateral undescended testes.* Drug Des Devel Ther, 2014. **8**: p. 2089-97.
32. Khramtsova, Y.S., et al., *The influence of mast cells on reparative regeneration of tissues characterized by various degrees of immune privilege.* Cell and Tissue Biology, 2016. **10**(5): p. 378-386.
33. Karakuş, S.C., et al., *The effect of hypothermia in a rat testicular torsion/detorsion model.* J Pediatr Urol, 2021. **17**(3): p. 291.e1-291.e8.
34. Qo, S., *[Mast cell induction to the mouse testicular interstitium].* Nihon Hinyokika Gakkai Zasshi, 1994. **85**(5): p. 747-52.
35. Majeed, S.K., *Mast cell distribution in mice.* Arzneimittelforschung, 1994. **44**(10): p. 1170-3.
36. Li, X., et al., *Transgenic Mice Expressing P450 Aromatase as a Model for Male Infertility Associated with Chronic Inflammation in the Testis.* Endocrinology, 2006. **147**(3): p. 1271-1277.
37. Nour, N. *Involvement of Activin and Follistatin in the Pathogenesis of Chronic Testicular Inflammation in Mice*. 2017.
38. Mendis-Handagama, S.M., et al., *Comparison of testis structure, function and thyroid hormone levels in control C57BL/6 mice and anti-mullerian hormone over expressing mice.* Histol Histopathol, 2010. **25**(7): p. 901-8.
39. Sayed, R.K.A., et al., *Retinoid-related orphan nuclear receptor alpha (RORα)-deficient mice display morphological testicular defects.* Laboratory Investigation, 2019. **99**(12): p. 1835-1849.
40. Bakhtiary, Z., et al., *Ethyl Pyruvate Ameliorates The Damage Induced by Cyclophosphamide on Adult Mice Testes.* International journal of fertility & sterility, 2016. **10**(1): p. 79-86.
41. Frungieri, M.B., et al., *Serotonin in Golden Hamster Testes: Testicular Levels, Immunolocalization and Role during Sexual Development and Photoperiodic Regression-Recrudescence Transition.* Neuroendocrinology, 1999. **69**(4): p. 299-308.
42. Mayerhofer, A., et al., *Histamine affects testicular steroid production in the golden hamster.* Endocrinology, 1989. **125**(4): p. 2212-4.
43. Anosa, V.O. and J.J. Kaneko, *Pathogenesis of Trypanosoma brucei infection in deer mice (Peromyscus maniculatus). Ultrastructural pathology of the spleen, liver, heart, and kidney.* Vet Pathol, 1984. **21**(2): p. 229-37.
44. Menezes, T.P., et al., *Pattern of protein expression in the epididymis of Oligoryzomys nigripes (Cricetidae, Sigmodontinae).* Cell and Tissue Research, 2018. **372**(1): p. 135-147.
45. Frungieri, M.B., et al., *Neuronal Elements in the Testis of the Rhesus Monkey: Ontogeny, Characterization and Relationship to Testicular Cells.* Neuroendocrinology, 2000. **71**(1): p. 43-50.
46. Schmid, N., et al., *Characterization of a non-human primate model for the study of testicular peritubular cells-comparison with human testicular peritubular cells.* Mol Hum Reprod, 2018. **24**(8): p. 401-410.
47. Souza, A.C.F., F.C. Santos, and D.S.S. Bastos, *Reproductive functions in Desmodus rotundus: A comparison between seasons in a morphological context.* 2018. **13**(10): p. e0205023.

48. Machado-Neves, M., et al., *Dietary Exposure to Tebuconazole Affects Testicular and Epididymal Histomorphometry in Frugivorous Bats.* Bull Environ Contam Toxicol, 2018. **101**(2): p. 197-204.
49. Dhyana, R., et al. *MICROANATOMICAL STUDIES ON THE TESTIS OF DOMESTIC PIG (Sus scrofa domestica)*. 2016.
50. Ohanian, C., et al., *Studies on the contractile activity and ultrastructure of the boar testicular capsule.* Reproduction, 1979. **57**(1): p. 79-85.
51. Kangawa, A., et al., *Histological Changes of the Testicular Interstitium during Postnatal Development in Microminipigs.* Toxicol Pathol, 2019. **47**(4): p. 469-482.
52. Pinart, E., et al., *Cytology of the interstitial tissue in scrotal and abdominal testes of post-puberal boars.* Tissue & cell, 2001. **33**(1): p. 8-24.
53. Wrobel, K.H., F. Sinowatz, and R. Mademann, *Intertubular topography in the bovine testis.* Cell Tissue Res, 1981. **217**(2): p. 289-310.
54. Goyal, H.O., *Morphology of the bovine epididymis.* American Journal of Anatomy, 1985. **172**(2): p. 155-172.
55. Abd-Elmaksoud, A., *Morphological, Glycohistochemical, and Immunohistochemical Studies on the Embryonic and Adult Bovine Testis.* . Dissertation, LMU München: Faculty of Veterinary Medicine, 2005.
56. Alkafafy, M., *Glycohistochemical, Immunohistochemical and Ultrastructural Studies of the Bovine Epididymis.* . Dissertation, LMU München: Faculty of Veterinary Medicine, 2005.
57. Elzoghby, I.M.A., et al., *Postnatal development of the epididymis in the sheep.* benha veterinary medical journal, 2014. **26**(1): p. 8.
